# Optimal Control of Transitions between Nonequilibrium Steady States

**DOI:** 10.1371/journal.pone.0082754

**Published:** 2013-12-26

**Authors:** Patrick R. Zulkowski, David A. Sivak, Michael R. DeWeese

**Affiliations:** 1 Department of Physics, University of California, Berkeley, California, United States of America; 2 Redwood Center for Theoretical Neuroscience, University of California, Berkeley, California, United States of America; 3 Center for Systems and Synthetic Biology, University of California San Francisco, San Francisco, California, United States of America; 4 Helen Wills Neuroscience Institute, University of California, Berkeley, California, United States of America; University of Nottingham, United Kingdom

## Abstract

Biological systems fundamentally exist out of equilibrium in order to preserve organized structures and processes. Many changing cellular conditions can be represented as transitions between nonequilibrium steady states, and organisms have an interest in optimizing such transitions. Using the Hatano-Sasa Y-value, we extend a recently developed geometrical framework for determining optimal protocols so that it can be applied to systems driven from nonequilibrium steady states. We calculate and numerically verify optimal protocols for a colloidal particle dragged through solution by a translating optical trap with two controllable parameters. We offer experimental predictions, specifically that optimal protocols are significantly less costly than naive ones. Optimal protocols similar to these may ultimately point to design principles for biological energy transduction systems and guide the design of artificial molecular machines.

## Introduction

Living systems are distinguished by their self-organization. Given the entropic driving force embodied in the second law of thermodynamics, creating and maintaining such organization requires staying far from equilibrium [1], typically by coupling to nonequilibrium gradients. For example, ATP-driven molecular motors (*e.g.*, kinesin) are forced away from equilibrium by cellular maintenance of a chemical potential difference between ATP and ADP [2], and the rotary 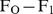 ATP synthase operates out of equilibrium due to cellular maintenance of an electrochemical gradient across the inner mitochondrial membrane [3]. For constant ATP and ADP concentrations, or constant membrane potential, the dynamics of an ensemble of such molecular motors will approximate a nonequilibrium steady state (NESS). Thus, biological systems are often better characterized as nonequilibrium steady states rather than equilibrium systems. Such NESS may change in response to changing environmental conditions. Given that selective advantage may be incurred by energetically-efficient operation, evolution may have sculpted biological components to interact so as to reduce the energy wasted during transitions between NESS. Accordingly, optimizing such transitions may offer insights into the design principles of biological systems and guide the creation of synthetic molecular-scale machines.

Much recent attention has focused on predicting optimal protocols to drive systems between equilibrium states with minimal expended work [4]–[10]. In particular, Ref. [4] proposes a linear response framework for protocols that minimize the dissipation during nonequilibrium perturbations of microscopic systems. This idea is developed further in [5] where the utility of Riemannian geometry suggested in [4] is exploited to find explicit optimal protocols for a paradigmatic colloidal particle model. Our ultimate aim is to extend the geometric framework of [4] to optimal transitions between steady states so that the tools utilized in [5] may be applied to more biologically relevant models.

In this article, we take a first step towards this goal by optimizing the Hatano-Sasa Y-value, a quantity similar to dissipated work, for the paradigmatic model system tested in [11] and analyzed in [12] with an eye towards experimental tests. We calculate closed-form expressions for both the geodesic optimal protocol and the optimal straight-line protocol and test these protocols numerically via a system of equations derived from the Fokker-Planck equation. Finally, we propose a regime of validity of our approximation based on this numerical work. By measuring the average work required to drive this system along either optimal or naive paths through control parameter space, our results can be tested experimentally in a straightforward way using existing experimental techniques.

## Results

### The model system and its inverse diffusion tensor

We consider a particle with spatial coordinate 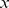 diffusing under Langevin dynamics subject to a one-dimensional harmonic potential, with equation of motion.
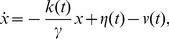
(1)


for Gaussian white noise 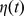.Here 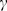 is the Cartesian friction coefficient, 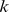 is the trap stiffness, 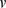 is the trap center velocity in the lab frame and 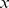 is the coordinate of the colloidal particle in the frame co-moving with the trap. The particle is initially in NESS due to constant trap velocity 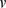.

As defined in [13], the Hatano-Sasa Y-value
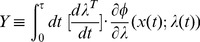
(2)


arises in NESS transitions when the control parameters 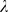 are changed rapidly compared to the system's relaxation timescale. Here 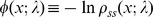 where 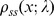 is the steady state probability distribution and 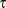 is the protocol duration.

In some simple cases this corresponds to the system ‘lagging’ behind the changing control parameters. For transitions between equilibrium states this measure reduces to the standard dissipation governed by the Clausius inequality [14]. This measure of irreversibility (2) obeys a significant NESS fluctuation theorem that has been experimentally observed in our particular model system [11]. We may derive an approximate scheme, exact in the linear response regime [15], for optimizing this Y-value during finite-time transitions between different nonequilibrium states.

The ensemble average of the Y-value is
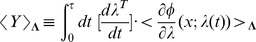
(3)


During the driving process, the system's probability distribution over microstates fundamentally depends on the history of the control parameters 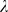, which we denote by the control parameter protocol 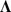. We assume the protocol to be sufficiently smooth to be twice-differentiable.

Applying linear response theory [4], [5], [15] and assuming that the protocol varies sufficiently slowly [4], we arrive at an expression for the average Y-value.
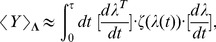
(4)


in terms of the control parameter velocities 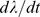 and the inverse diffusion matrix 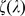 with entries
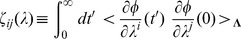
(5)


The angle brackets 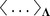 represent an average over noise followed by a stationary state average over initial conditions using the distribution 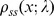. Note that, if 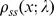 is the equilibrium distribution, the inverse diffusion tensor of [5] is recovered.

In general, the detailed balance condition is violated in NESS and so the matrix 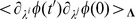 may be asymmetric. Eq. (4) shows the use of Eq. (5) (specifically its symmetric part) as a metric tensor is not precluded. However,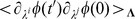 is not a covariance matrix and so a general proof of positive-definiteness is lacking [4]. These considerations do not affect the model considered here but future work is needed to address this issue for the general case.

The steady-state distribution is given by 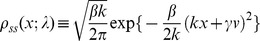 [11], [12]. The inverse diffusion tensor is given by
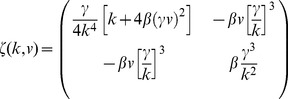
(6)


### Optimal protocols

Though one can write down the geodesic equations for the metric [Eq. (6)] in the 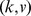 coordinate system, more insight is gained by finding a suitable change of coordinates. A direct calculation of this metric's Ricci scalar yields 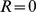, demonstrating that the underlying geometry is Euclidean [16].

With the coordinate transformation 
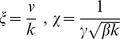
, the line element is
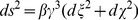
(7)


**Figure 1:**
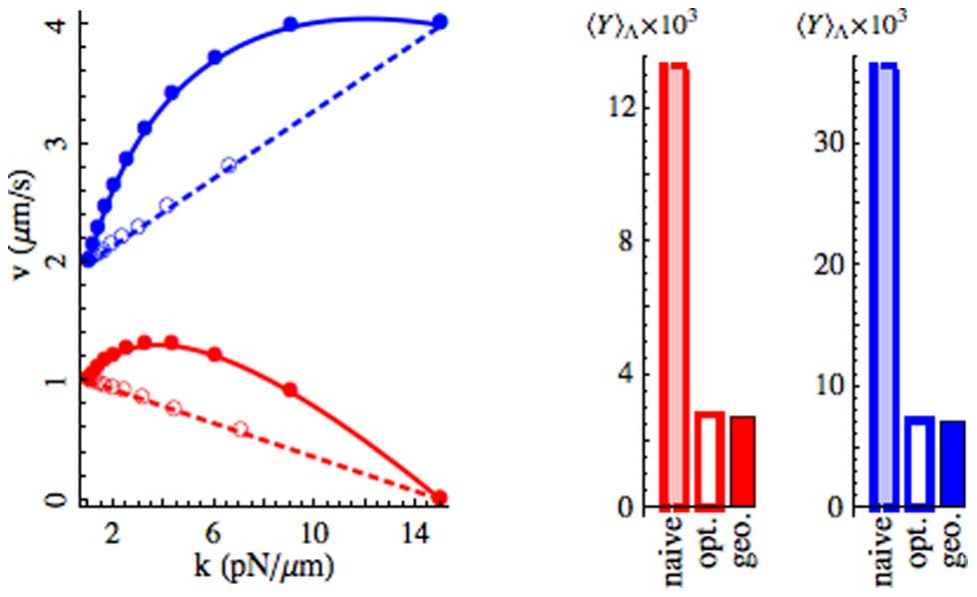
Geodesics describe protocols that outperform naive (constant-speed) straight-line paths in parameter space. Geodesics between fixed pairs of points in the 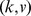-plane and accompanying straight-line protocols are pictured in (a). The filled circles represent points separated by equal times. The open circles correspond to the optimal parametrization along the respective straight path. All mean Y-values were calculated using the Fokker-Planck system, Eq. (15). Here, 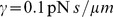 and 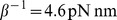 to approximate the experiments of Ref. [11]. The protocol duration is chosen to be 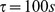 to ensure that the relative error 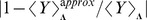 is less than 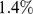 for all protocols. Protocol endpoints were selected for experimental accessibility [21]. The relative performance of naive straight-line, optimal straight-line, and geodesic protocols are summarized in (b).

In this coordinate system, geodesics are straight lines of constant speed. By the covariance of the geodesic equation, the optimal protocols are given by
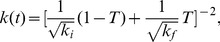
(8a)

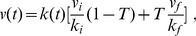
(8b)where 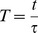. Sample optimal protocols are pictured in Fig. 1.

### Optimal straight-line protocols

In the absence of any particular information about the system's dynamical properties, a naive control strategy would change the control parameters at a constant rate, producing a straight line in control parameter space. The inverse diffusion tensor approximation [Eq. (4)] provides a recipe for choosing both a potentially nonlinear path through control parameter space, as well as a time-course along that path. The inverse diffusion tensor formalism can alternatively be used to optimize the time-course along a straight-line control parameter path. Such a protocol provides a benchmark against which an optimal protocol [Eq. (8)] can be compared. For the model considered here, we will find that an optimal straight-line protocol can be substantially better than the most naive (constant-speed) straight-line protocol. Furthermore, straight-line protocols are relatively straightforward to test experimentally.

**Figure 2:**
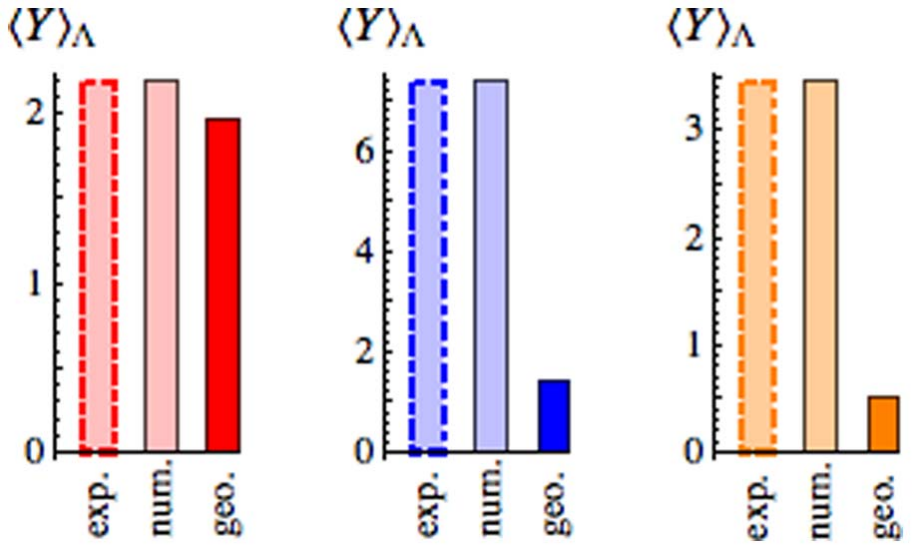
Optimal protocols outperform constant-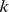 protocols tested in Ref. [11]. Experiment 1 (left, red) used a quarter-sine wave protocol to vary the trap speed; Experiments 2 (middle, blue) and 3 (right, orange) used an inverted three-quarters sine wave. Specifically, 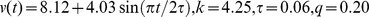 for Experiment 1, 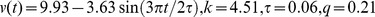 for Experiment 2, and 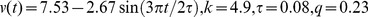 for Experiment 3. Here, velocity is measured in 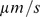, 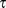 is the protocol duration measured in 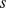, 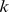 is the trap stiffness measured in 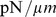, and 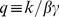 is measured in 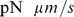. The Y-value for these protocols (light color bar) and for the optimal protocols (solid color bar) were obtained numerically assuming 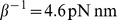 (red), 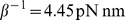 (blue), 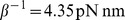 (orange) respectively. These effective temperatures were chosen to give the best match between experiment and numerical calculation, and may differ from room temperature (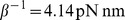) because of local heating by the optical trap [22]. We predict a significant reduction in Y-value for optimal protocol driving under the conditions of the three experiments described in Ref. [11].

When 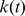 is held fixed, a straightforward application of variational calculus demonstrates that a straight-line protocol in 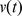 is *exactly* optimal and agrees with the predictions of the linear response approximation [Eq. (4)]. In Ref. [11], the average Y-value was measured for three distinct experimental trials involving protocols with constant 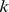. As summarized in Fig. 2, the optimal protocol, namely the naive straight line in the case of constant 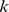, shows significantly reduced Y-value compared with the protocols used in each experimental trial. However, in terms of testing the performance of the optimal protocols [Eq. (8)], 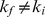 is the more general case.

As in the case of finding globally optimal protocols, the problem of finding optimal straight line protocols simplifies dramatically in 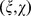 coordinates. Using Eq. (7), we find.
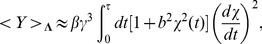
(9)


for
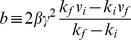
(10)


The Euler-Lagrange equation implies
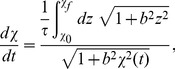
(11)


which determines an implicit expression for 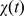:
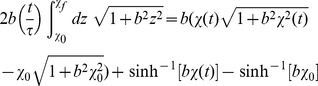
(12)


The relation 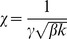 determines an implicit expression for 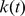, and hence for 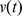.

### Computing the Y-value numerically

We validate the optimality of the geodesics [Eq. (8)] and compare with optimal straight-line protocols by calculating the average Y-value directly by integrating in time the Fokker-Planck equation describing the dynamical evolution of the particle probability distribution [12],
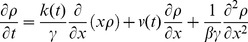
(13)


In full generality, the mean Y-value as a functional of the protocol 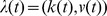 is
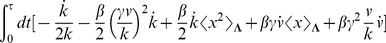
(14)


Here angled brackets denote averages over the nonequilibrium probability density 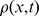.

By integrating Eq. (13) against 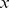 and 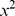, we find a system of equations for relevant nonequilibrium averages:
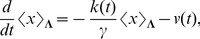
(15a)

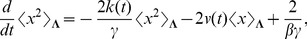
(15b)


supplemented by initial conditions
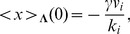
(16a)

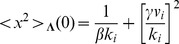
(16b)


Note that for a more complex system the first and second moments 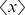 and 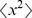 are not sufficient to characterize the probability distribution, but time-dependent solutions are still accessible through standard (but more computationally intensive) numerical integration of the full Fokker-Planck equation (13) [17].

We solve these equations numerically and compare the performance of optimal straight lines against geodesics [Eq. (8)] and naive (constant-speed) straight-line protocols in Fig. 1. We selected endpoints and physical constants based on those used in the experiments of Ref. [11] which may be found in the caption of Fig. 2. In this near-equilibrium regime the inverse diffusion tensor approximation produces small relative error in Y-value. Though there is only a marginal difference in performance between the optimal straight-line protocol and the geodesic for both sets of endpoints, there is a substantial benefit in using either over the naive straight line protocol.

### The inverse diffusion tensor arises naturally from the Fokker-Planck equation

If we neglect terms involving second- and higher-order temporal derivatives (an alternative near-equilibrium approximation), we obtain an approximate solution to the Fokker-Planck system:
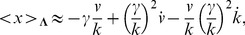
(17a)

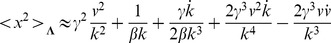
(17b)


Substituting this into the expression for the mean Y-value [Eq. (14)], we recover Eq. (28). The argument above suggests that the emergence of the inverse diffusion tensor from the Fokker-Planck equation may follow from a perturbation expansion in small parameters [5].

## Discussion

We have taken the first step towards extending the geometric framework for calculating optimal protocols presented in [4], [5] to systems relaxing to NESS. As energy-transducing biological systems are more faithfully described by NESS than by equilibrium statistics, this brings recent theoretical developments closer to the behavior of *in vivo* biological systems.

Using a linear response approximation, we found the optimal mean Y-value for a model system of a colloidal particle (initially in NESS) dragged through solution and subject to a time-dependent harmonic potential. We took as our control parameters the velocity and spring constant of the harmonic potential. As in [5], tools from Riemannian geometry revealed a useful coordinate transformation which greatly simplified the construction of optimal straight-line protocols as well as geodesic protocols. These optimal protocols were tested numerically and the small relative error in the Y-value approximation for experimentally relevant choices of parameters is encouraging.

Our predictions may be tested experimentally with existing hardware and methods. In Ref. [11], the authors report on experiments performed with micron-sized polystyrene beads in solution. The harmonic potential is created by superposing the foci of two counterpropagating laser beams. The location of this trap was translated using a steerable mirror. The velocity 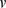 of the trap location was altered by changing the mirror's angular rate of rotation, and the trap stiffness 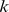 can be manipulated by dynamically changing the intensity of the laser beam [18] or by passing the laser beam through a polarization filter and dynamically changing the polarization of the laser beam. Force is inferred from the rate of change of the momentum of light measured by position-sensitive photodetectors. Comparison of the average work incurred during different protocols would provide an experimental test of the optimal protocols predicted in this manuscript.

Using the inverse diffusion tensor approximation in general allows us access to the full power of Riemannian geometry in calculating optimal protocols. However, such experimental tests can assess the range of validity of the approximation. Our alternate derivation of the inverse diffusion tensor via a “derivative-truncation” expansion [5] suggests a greater robustness of the approximation.

In this paper we provide concrete theoretical predictions for experiments – specifically, we find that geodesics, optimal straight-lines, and naive straight-line protocols all are substantially more efficient than the protocols tested in Trepagnier, *et al.* Moreover, we demonstrate that for simultaneous adjustment of 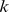 and 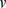, optimal straight-line protocols can perform substantially better than naive (constant-speed) straight-line protocols. The necessary methodology and experimental apparatus are well-established [11] to not only verify our predictions but to push beyond the near-steady-state regime.

Given the greater generality embodied by the extension to NESS, and the accuracy of this approximation for a standard model system, optimal driving protocols derived in this framework promise greater applicability to models of biomolecular machines. Nevertheless, important hurdles remain: our model system experiences forces linear in position and has a steady-state distribution differing from the equilibrium one only in its average displacement. Molecular machines feature nonlinear force profiles, potentially nontrivial steady-state distributions, and often operate far from equilibrium. Thus our comparatively simple theoretical framework may need further elaboration to address the dynamics and efficiency of molecular machines with reasonable fidelity.

Furthermore, the relatively simple model system we treat in this manuscript represents a new frontier for the analytical solution of optimal protocols under the inverse diffusion tensor approximation. For significantly more complicated models of greater biological interest, a simple general approach (in lieu of a search for an analytical solution) would be a fully numerical method, involving the calculation of the inverse diffusion tensor at a grid of points in control parameter space, analogous to the approach in [19].

Finally, there remains the important open question of what quantity or quantities are to be optimized in faithful models of biological processes. In this paper, we made the choice of optimizing the Y-value which has been experimentally studied in this particular model system [11] and may be optimized by the same geometric framework as in [4]. These qualities were advantageous to begin a clear and mathematically tractable first step towards optimization of steady state transitions.

However, it is possible and perhaps likely that a properly defined average dissipated heat will be the biologically relevant quantity to optimize rather than the Y-value. We anticipate that a geometric approach to optimization will be applicable to these more general systems and notions of heat production in a relevant regime of parameter values and protocol durations. However, a more general construction will have to take into account the so-called housekeeping heat [13], [20] which is generated in maintaining the steady state at given control parameter values. Future work is needed to address these issues properly.

## Methods

Our model consists of a particle with spatial coordinate 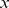 diffusing under Langevin dynamics subject to a one-dimensional harmonic potential, with equation of motion
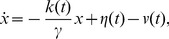
(18)


for Gaussian white noise 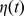 satisfying
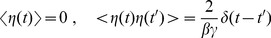
(19)Here 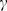 is the Cartesian friction coefficient, 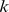 is the trap stiffness, 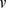 is the trap center velocity in the lab frame and 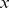 is the coordinate of the colloidal particle in the frame co-moving with the trap. The particle is initially in NESS due to constant trap velocity 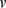.

As defined in [13], the Hatano-Sasa Y-value is given by
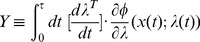
(20)Here 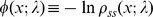 where 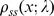 is the steady state probability distribution and 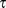 is the protocol duration.

The ensemble average of the Y-value is
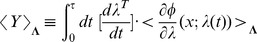
(21)


Applying linear response theory [4], [5], [15] and assuming that the protocol varies sufficiently slowly [4], we arrive at an expression for the average Y-value
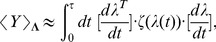
(22)


in terms of the control parameter velocities 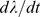 and the inverse diffusion matrix 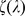 with entries
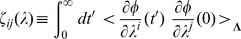
(23)


The angle brackets 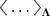 represent an average over noise followed by a stationary state average over initial conditions using the distribution 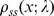.

The steady-state distribution is given by 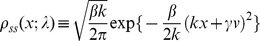 [11], [12]. The parameter space derivative of 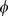 is given by
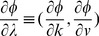
(24a)

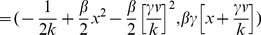
(24b)


In order to calculate the time correlation functions in Eq. (23), we solve Eq. (18) for constant 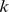 and 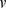, giving
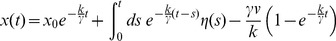
(25)


Recalling that 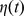 is Gaussian noise, Eq. (25) implies
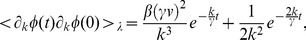
(26a)

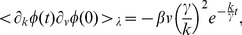
(26b)

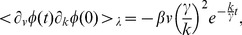
(26c)

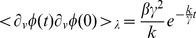
(26d)


Integrating over time yields the inverse diffusion tensor:
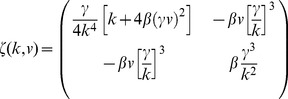
(27)


The line element corresponding to the metric in Eq. (27) is
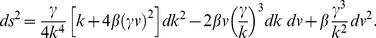
(28)


To find the explicit coordinate transformation making the Euclidean geometry manifest, we write the line element as
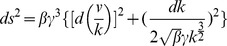
(29)


This suggests the coordinate transformation 
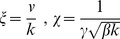
, so that
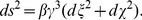
(30)


In this coordinate system, geodesics are straight lines of constant speed. To find optimal protocols in 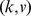 space, one simply transforms the coordinates of the endpoints into 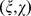 space, connects these points by a straight line, and uses the inverse transformation to map the line onto a curve in 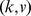 space. This follows from the invariance of the geodesic equation [16]. Explicitly, the optimal protocol joining 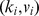 and 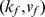 is
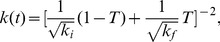
(31a)

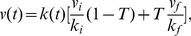
(31b)where 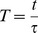.

We validate the optimality of the geodesics [Eq. (31)] numerically via the Fokker-Planck equation [12],
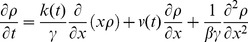
(32)


In full generality, the mean Y-value as a functional of the protocol 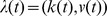 is
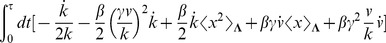
(33)Here angled brackets denote averages over the nonequilibrium probability density 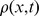.

By integrating Eq. (32) against 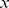 and 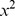, we find a system of equations for relevant nonequilibrium averages:
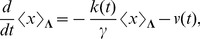
(34a)

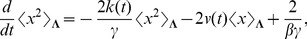
(34b)


supplemented by initial conditions
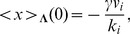
(35a)

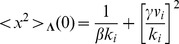
(35b)

